# Evaluating effects of aging on dog olfactory performance

**DOI:** 10.1007/s11357-025-01905-1

**Published:** 2025-11-03

**Authors:** Lane I. Montgomery, Sarah Krichbaum, Jeffrey S. Katz, Lucia Lazarowski

**Affiliations:** 1 Department of Psychological Sciences, Auburn University, 226 Thach Hall, Auburn, AL 36849 USA; 2 Canine Performance Sciences, College of Veterinary Medicine, Auburn University, Auburn, AL 36849 USA

**Keywords:** Dog, Aging, Olfaction, Cognition, Training

## Abstract

Aging is a dynamic process across a dog’s lifespan, with age-related changes impacting how dogs function in their companion and working roles. However, the impact of aging on key sensory modalities, such as olfaction, is not well understood. The current study aimed to characterize age-related changes in canine olfactory abilities via an adaptation of the Natural Detection Task. Data were collected on 65 dogs aged 5 years or older, with 44 dogs completing all components of the task. We found age-related decrements in olfactory performance, but only under certain conditions and with differential effects as a function of training level. Age also affected whether dogs completed the entirety of the task, indicative of non-olfactory (e.g., physical or motivational) factors. Additionally, dogs completed the Cylinder Reversal Task (CRT) to determine whether olfactory changes were correlated with cognitive changes. We found no age-related changes in CRT performance and no significant associations between cognitive and olfactory task performance. These findings indicate that dogs’ olfactory capabilities decrease with age but, critically, training experience may have protective effects against age-related declines, and effects may only be apparent under certain conditions. Future studies should continue to consider olfaction when studying aging impacts upon the dog.

**Supplementary information:**

The online version contains supplementary material available at 10.1007/s11357-025-01905-1.

## Introduction

The effects of aging on dog quality of life are important to understand, as aging can have significant impacts on daily function and welfare. Behavioral and cognitive changes due to the aging process are well documented in dogs [1], and large-scale studies have sought to characterize factors that influence aging and ameliorate its effects (e.g., The Dog Aging Project [2]). However, the focus of this body of research has largely been directed toward health and cognition, with less attention paid to the effects of aging on sensory domains such as olfaction.

Age-related declines in olfactory function have been shown in various species, including humans [3, 4], mice [5], and rats [6]. However, while dog olfaction is an increasing area of research [7], few studies have investigated decrements in canine olfaction related to aging [8–10]. Characterizing age-related changes in dogs’ olfactory capabilities is valuable, given the centrality of olfaction to the dog’s perceptual experience [11] and, in turn, welfare [12]. Furthermore, the use of dogs trained for odor detection continues to grow, and understanding how olfaction changes with age is critical to managing the effectiveness of aging detection dogs. Addressing this area may also be valuable because olfactory dysfunction is predictive of cognitive impairments and neurodegeneration in humans [13], indicating that the use of such assessments in tandem could give a more comprehensive picture of aging declines in dogs.

Various measures have been designed for rapid assessment of age-related cognitive and behavioral changes in dogs, including: the executive function task battery [14]; the dog cognitive development battery [15]; the neophobia and spatial memory battery [16]; serial reversal learning [17]; and spatial working memory [18]. However, most measures of olfactory capability require substantial training and time, limiting their utility for rapid evaluations of olfactory function [19–21]. Further, performance on such tests is likely influenced by non-olfactory factors such as trainability, motivation, and attention, making it difficult to isolate olfactory ability. To address these concerns, experimenters designed the Natural Detection Task (NDT) to evaluate olfactory abilities rapidly without the requirement of prior training [22]. However, even within studies that have used this task to examine aging [9, 10], conclusions are limited by the use of arbitrary age categorizations, which can result in inaccurate findings that do not capture the continuous nature of dog aging [23]. Although this approach is not uncommon in aging studies, nonbiological age categorization may influence findings by increasing the risk of type one error [23].

The current study aimed to examine age-related changes in olfactory function in dogs ranging from mature adult to geriatric ages. To this end, dogs completed a variation of the NDT, a task that requires dogs to locate a hidden piece of food under varying levels of concealment and resulting odor availability [22]. In addition to typical conditions of the NDT, which measure odor detection sensitivity, the current study included a condition to assess dogs’ ability to detect the target odor among an extraneous background odor. This condition was added to evaluate how other aspects of olfactory function, in this case, selectivity [24], are impacted by the aging process. Selectivity was chosen due to its applications for detection dogs, as detection of a target odor rarely occurs in isolation without potential interference of background or extraneous odors. Interference from other odors may occur at a neural level [24], significantly altering the perceptual experience of the dog. However, it is not known whether aging impacts different aspects of olfactory ability in varying ways.

Dogs also completed the Cylinder Reversal Task (CRT) to examine relationships between olfactory performance and cognition. This task was chosen due to its (1) ability to rapidly evaluate inhibitory control and (2) established link with symptomology of Canine Cognitive Dysfunction Syndrome (CCD), an age-related neurodegenerative disorder in dogs [14]. Additionally, validated psychometric questionnaire (PANAS [25], CCDR [26]) responses were collected from owners concerning behavior and cognitive function to replicate previous age-related findings [27, 28]. These questionnaires allowed verification that the age range captured in the current study would enable anticipated effects of aging to be identified.

We hypothesized that, as with other species [4–6], olfactory detection abilities would decrease with increasing age. Additionally, we hypothesized that performance in the CRT would be correlated with olfactory task performance, similar to findings of associations between olfaction and cognition in humans [13].

## Methods

### Subjects

Sixty-five dogs (*F* = 41; *M* = 24) aged 5 years or older (*M* = 7.78 years; SD = 2.5; age range = 5–15) participated in the study. The age of the dogs was restricted to ensure representation of aging canines without the influence of early developmental processes. Both purebred and mixed-breed dogs participated in the study (see Supplementary Materials). The sample included both companion dogs (*n* = 56) and trained detection dogs (*n* = 9) from a detection dog breeding and training program (Auburn University Canine Performance Sciences). All methods were approved by the Auburn University Institutional Animal Care and Use Committee (#2022–5104) and conducted in accordance with all relevant guidelines and regulations.

### Natural detection task

#### Odors

Consistent with previous studies using the NDT, the target odor stimulus was a piece of food (Pet Botanics® Training Rewards Beef Flavor), selected as a stimulus that should elicit a natural and spontaneous response from an untrained dog [22]. In instances where the dog had dietary limitations or refused to ingest the provided food, PureBites® Lamb Liver Freeze Dried Dog Treats or an owner-provided alternative was used. In these cases, experimenters ensured that treat sizes were consistent across testing. Cotton rounds immersed in coffee grounds were used for the extraneous odor condition in the NDT.

#### Odor presentation

**Fig. 1:**
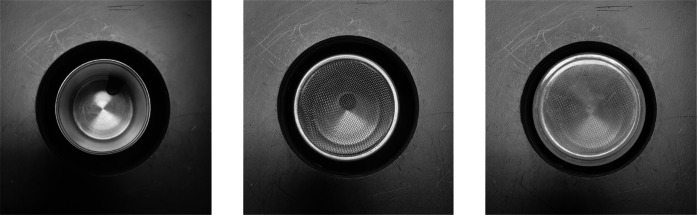
Canister cap types (left to right: open top, mesh cap, plastic cap)

Odors were presented in purpose-built odor presentation boxes (20 cm × 20 cm × 20 cm). The boxes were fitted with removable lids and designed to hold metal canisters that contained the target stimulus. The metal canisters could be fitted with either a mesh cap or a solid cap (see Fig. 1). A hole in the top of the lid of the box allowed the dog to sniff the contents without visual or physical contact with the canister inside the box.

#### Experimenter roles

Throughout all phases of the NDT, Experimenter 1 (E1) handled the dog and judged the dog’s responses. E1 was blind to target location during trials in which there were multiple boxes, so that interpretation of dog behavior was not biased by their knowledge of the target location. Experimenter 2 (E2) set up and timed test trials, confirmed responses, and hand coded responses. E2 wore gloves to minimize contamination when handling materials. E1 remained the same across all dogs to provide consistency in judging dogs’ responses.

#### Acclimation and warmup phase

Prior to the beginning of testing, all dogs were allowed to explore the testing space freely for at least 3 min. After the acclimation period, owners were instructed to sit approximately 1 m behind the testing setup and asked not to provide any cuing information to the dog or E1 during trials. Testing began with a warmup phase to establish that the dog could locate the food in the search task and the ability of E1 to detect an orienting response for that dog. An orienting response was defined as a change in dog behavior at a box that was different or more extreme than the typical behavior in the trial, indicating that the dog had located the target. For example, pawing at the box or chewing on the lid. The warmup phase consisted of four stages, described below, first establishing that the dog would engage with the box, then that the dog would sniff the box for a reward, next that the dog would elicit an orienting response on a singular box, and lastly demonstrating that the dog showed a clear orienting response when multiple boxes were present. If, during any stage, the dog timed out (i.e., did not provide a response within the maximum trial time) three times, the warmup phase ceased and testing moved on to the CRT, because the dog had not demonstrated a willingness to engage with the odor task or E1 was unable to distinguish a response.

In the first stage, a singular odor box was placed 1 m away from the dog. The lid from the box was removed, and a singular treat was placed within the box by E2. The treat was not placed within a canister at this time, so that the dog could access the treat independently. The dog was then released from the start line by E1 and could retrieve the treat from the open box. This stage was performed to establish an association between the box and a food reward and to confirm that the dog was comfortable interacting with the box. If the dog would not retrieve the treat within 30 s, then the trial was marked as a timeout. If the dog required the experimenter to remove the treat from the apparatus or significant encouragement (i.e., any intervention by E1 greater than gesturing toward the box and calling the name of the dog) to engage with the apparatus, then the trial was marked incorrect. If the dog retrieved the treat from the box with minimal interference by E1, then the trial was marked correct. Once the dog had completed three correct trials, the dog proceeded to stage two.

In the second stage, a treat was placed in an open metal canister within the box, and the box lid was placed on the odor box. When the dog sniffed the top of the odor box, E1 praised the dog, removed the lid, and provided the dog with access to the treat. Through this process, the dog was shown that sniffing the box resulted in reinforcement. If the dog would not sniff or otherwise engage with the box within 30 s, then the trial was marked as a timeout. If the dog required significant encouragement to engage with the box, the trial was marked incorrect. If the dog sniffed the box and took the treat once the lid was removed, the trial was marked correct. Three trials of this stage were completed regardless of the dog’s performance.

The third stage was the same as the second stage in terms of apparatus and setup. However, in stage three, the dog had to provide an orienting response to receive the treat. A trial was marked as a timeout if the dog did not engage with the box within 30 s. A trial was considered incorrect if the dog required significant encouragement to show an orienting response and correct if the dog provided an orienting response with minimal to no encouragement. A dog was required to perform three trials correctly (non-consecutively) to move on to the next stage.

In the fourth and final stage of the warmup, two boxes were placed 1 m apart and 1 m from the start line. Between each trial, E1 led the dog into an adjacent holding room so that neither the dog nor E1 observed the setup of the next trial. The door to the holding room remained open such that the dog could maintain visual contact with their owner during this time without observing the setup. From this point forward, boxes were cleaned between trials to prevent odor cues beyond the target odor from informing the target location. E2 would then move behind an opaque barrier (to prevent unintentional cuing) and alert E1 that the trial was ready, at which point E1 would walk toward the boxes with the dog on a loose leash. E1 would attempt to walk the dog through the boxes left to right, so that the dog did not rely upon a locational bias for its responses. During these trials, E1 gestured toward the boxes as needed to encourage the dog to sniff. If the dog showed an orienting response at a box, E1 would call out the location (i.e., “left,” “right”) to E2. E2 would reply “yes” or “no” based upon whether the target was present at that location. If the location was correct, E1 would remove the box lid, praise the dog, and allow them to access the treat. For incorrect locations, if the dog had sampled both boxes, the trial would end at that time, with the dog being guided back to the holding room with no verbal or physical indication of their performance. If the dog had not sampled both boxes, the dog was allowed to sample the unsampled box before the end of the trial. If the dog showed an orienting response on that box, E1 could call the additional response and reward the dog. By doing so, the dog could demonstrate that they had found the odor even if they provided an incorrect response in their first attempt. If E1 had presented both boxes to the dog and the dog had not provided an orienting response, E1 would stand with the dog on a loose leash and allow them to sample at will. If the dog had not made a choice within 30 s, the trial was marked as a timeout. The dog was required to complete four trials in this stage, with the target occurring twice in each location. Accuracy did not impact the ability of the dog to move forward in test trials. However, if the dog received three timeouts, then it would not complete the NDT and would move straight to the CRT.

#### Natural detection task conditions

After the warmup phase, dogs completed an adapted version of the NDT. The task was altered per original author recommendations for fewer levels [22], and with the addition of an extraneous odor condition, to better capture the behaviors of interest. Notably, the aforementioned warmup phase was included (increasing the learned component of the task but allowing the dog to achieve successful target localization before test trials) and the definition of a response was altered from Polgár et al. [22]. During this task, four odor boxes were placed 1 m from the start line and 0.8 m apart in a semicircular fashion. One of the four boxes was baited with a canister containing the food target. The type of canister was condition-dependent, with the task consisting of four conditions: an open condition, two odor detection threshold conditions, and one extraneous odor condition. In the open condition, a food reward was placed in the metal canister with no cap to establish the ability of the dog to detect the target with no interference. Similar to Polgár et al. [22], in the two threshold conditions, caps of the metal canisters were varied in order to modify odor availability. One condition consisted of a mesh lid to partially restrict the amount of odor escaping, and the other consisted of a plastic cap to completely conceal the container (see Fig. 1). In the extraneous odor condition, a second open canister (i.e., no lid) with a coffee-scented cotton round (the extraneous odor) was also present in the target box alongside the canister containing the target (with no lid). The contents of the empty boxes matched those of the target box, minus the presence of the food target (i.e., canister, corresponding lid for that condition, and extraneous odor for extraneous odor condition).

The open condition occurred first for each dog, serving as a baseline before task difficulty increased. The order of the other three conditions was counterbalanced across dogs so that task difficulty did not increase systematically with session length and potential fatigue. Within each condition, four trials were pseudorandomized such that each possible location served as the correct location once, and the last location of one condition was not the first location of the next. Between trials, the dog and E1 went into the holding room, and the boxes were cleaned by E2. The procedure for each condition followed the same protocol as stage four of the warmup phase, with only two differences. First, E1 would call the position number (1–4) instead of “left” or “right” to indicate the proposed location of the target. Second, dogs had 60 s to complete a trial instead of 30, reflecting the increased number of boxes to sample. If the dog did not make a choice within 30 s, E1 could re-present the boxes to the dog (i.e., walk the dog past the boxes from left to right again and gesture to them as needed). If the dog had not made an orienting response within 60 s, the trial was marked as a timeout. The dog was still allowed to sample unsampled boxes after an incorrect response and could do so until all boxes were sampled. If the dog received three timeouts within a condition, then the dog would complete three motivation trials. The setup and scoring of the motivation trials were the same as stage one of the warmup phase. The motivation trials were conducted to re-motivate the dog if they had lost interest during the task. Once this was completed, dogs resumed the task with the next trial. If the dog received three timeouts on the subsequent condition of the task, then the NDT was terminated, and the dog moved onto the CRT.

#### Dependent measures

Dogs that only performed part of the task were excluded from analyses (other than the task completion analysis), as failure to complete the task could represent motivational issues impacting performance rather than the variables of interest. The dependent variables included task completion (yes or no), a numerical response score for each condition based upon whether the first orienting response for each trial was correct (0–4), an overall response score, which was a summation of the response scores received for the individual conditions of the task (0–16), and an average accuracy, hit rate, and false alert rate for each condition and the task as a whole. Accuracy, hit rate, and false alert rate were calculated for each trial of each condition according to traditional scoring of these measures in an operational detection context [29]. Additionally, a correct rejection was considered not responding on a box that did not contain the target. Accuracy was calculated as the total number of hits and correct rejections divided by the total number of hits, correct rejections, false alerts, and misses. Hit rate was calculated as the total number of hits divided by the total number of hits and misses. False alert rate was calculated as the total number of false alerts divided by the total number of false alerts and correct rejections. Scores were averaged across trials to generate an average accuracy, hit rate, and false alert rate for each condition. All scores were based upon the first sampling of each box.

### Cylinder reversal task

#### Testing conditions

In this task, a plastic, open-ended cylinder attached horizontally to a wooden board was placed 1 m away from the dog. There were three phases to the task: familiarization, inhibitory control, and reversal (explained below). During the CRT, E1 set up trials and recorded data. E2 handled the dog, unless the dog showed fear or aggression in response to having its position adjusted by an unfamiliar person, in which case the owner handled the dog. At the start of each trial, E1 would call the dog’s attention (i.e., “[Dog name], look”) as necessary, then place the treat within the cylinder. Once the treat was placed within the cylinder, E1 would say “okay” and E2 released the dog from the start line. For each phase, the dog had a maximum of 15 trials to finish the phase. If the dog did not meet criteria within that time, the task was terminated. For each phase, a trial was considered a timeout if the dog had not engaged with the cylinder for at least 15 s. During a timeout trial, E1 could lure the dog to retrieve the treat. Timeouts were denoted separately from incorrect trials, but still were counted as trials in the data (i.e., considered in calculations of criteria). If a dog received three timeouts within a phase, then the task was terminated. If a dog made an incorrect response, the trial continued until the treat was retrieved or a timeout occurred.

#### Familiarization phase

In the familiarization phase, an opaque covering was placed over the cylinder. Trials occurred in the format described above. E1 counterbalanced the side through which they placed the treat into the cylinder across dogs. If a dog required multiple trials to complete this phase, the side through which E1 placed the treat was alternated to prevent a side bias from forming before direction-based testing began. The trial was considered correct if the dog retrieved the treat without touching the cylinder exterior and incorrect if the dog touched the exterior of the cylinder before retrieving the treat. This rule excluded instances in which the dog brushed against the side of the cylinder while retrieving the treat. The dog was required to get one trial correct to move forward to the next phase. The side through which the dog retrieved the treat on the correct trial was considered the “preferred side” for the next phase of the task.

#### Inhibitory control phase

In the inhibitory control phase, the opaque covering was removed from the cylinder so that it was transparent. The preferred side of the cylinder, as established in the familiarization phase, was left open while the unpreferred side was covered with a clear plastic disk. Trials occurred in the format described above. All treats were placed into the cylinder through the same side, as there was only one open end to the cylinder. The trial was considered correct if the dog retrieved the treat without touching the cylinder exterior and incorrect if the dog touched the exterior of the cylinder before retrieving the treat. The dog was required to get four of the last five trials correct (sliding block) to move forward to the next phase.

#### Reversal phase

In the reversal phase, the cylinder remained transparent but was rotated so that the preferred side was blocked and the unpreferred side was open. Trials occurred in the format described above, and treats were placed in the cylinder through the singular open side. A trial was considered correct if the dog approached the open side first. A trial was considered incorrect if the dog approached the closed side first, meaning it went to the side that was open in the previous phase. The dog was required to perform four of the last five trials correctly (sliding block) to finish the phase and complete the task.

#### Dependent measures

The dependent variables for this task included task completion (yes or no), percentage of trials with cylinder touches during the reversal phase (per [14]), and a score for each phase (familiarization range = 1**–**15; inhibitory control and reversal range = 5**–**15). If a dog failed to meet criteria in the maximum number of trials for a phase, that phase received a score of 16.

### Questionnaire

The questionnaire contained dog characteristic questions (e.g., age, sex, breed, neuter status), a question asking owners to report dog training history on a scale from 1 (no training experience) to 4 (advanced training), questions concerning prior cognitive task experience, the Positive and Negative Activation Scale (PANAS [25]), and the Canine Cognitive Dysfunction Rating Scale (CCDR [26]).

The PANAS is a validated assessment of dog responses to positive and negative stimuli, consisting of 21 items on a five-point Likert-type scale to produce scores on five subscales (Negative Activation and Overall Positive Activation, the latter further broken down into Energy and Interest, Persistence, and Excitability). Higher subscale scores represent higher levels of the trait [25].

The CCDR consists of 13 items on a five-point Likert-type scale which assesses behavioral abnormalities associated with age. These items are summed to give a level of cognitive impairment from normal to diagnosable levels of CCD [26].

### Data analysis

First, as several subjects had previous experience with the cylinder task from other studies (*n* = 15), independent sample *t*-tests were conducted to examine the effect of previous task experience on each of the dependent variables of the CRT. If performance differed between dogs with and without past cylinder task experience, subsequent analyses would be performed separately for each group for these measures. Generalized linear models (GLMs) were conducted with a Poisson distribution (except for NDT completion scores, for which a binomial distribution was used) to examine effects of age, sex, training level, and all two- and three-way interactions on each of the NDT and CRT dependent variables. Nonsignificant interactions were removed stepwise. Training level was examined as a variable of interest based on evidence that training may have neuroprotective effects against aging [27] and was therefore included as a covariate in the models. A one-way ANOVA was conducted to examine the effect of condition on overall response score to determine whether there were differences in difficulty between task conditions as presumed. In addition, Spearman correlations were conducted between the dependent measures of the NDT (overall response score and for each condition) and CRT (trials to criteria for each phase) to identify associations between the processes utilized in each task.

## Results

### Natural detection task

**Fig. 2:**
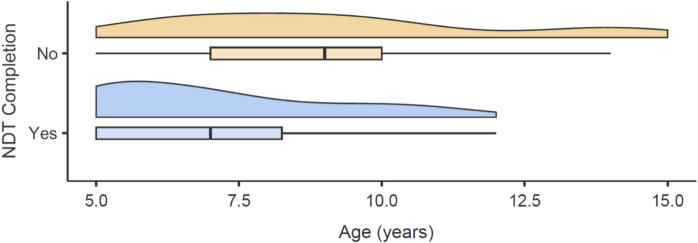
Effect of age (in years) on NDT completion. “No” on the y-axis represents dogs that did not complete the entire NDT (*n* = 21), while “yes” on the y-axis represents dogs that did complete the entirety of the NDT (*n* = 44)

Twenty-one dogs failed to complete at least one phase of the task (nine during warmups, 12 during the main task) and were therefore removed from all NDT analyses except for NDT completion, with 44 dogs remaining overall. There was a significant effect of both age and training level on NDT completion, such that older dogs were less likely to complete the NDT (*z* =  − 2.27, *p* = 0.023; see Fig. 2) and dogs with greater levels of training were more likely to complete the NDT (*z* = 2.75, *p* = 0.006).

For accuracy, hit rate, and false alert rate, 42 subjects had complete data for analyses, as two dogs’ sessions had video errors that prevented scoring of responses. Interrater reliability was calculated for accuracy for 20% of subjects (*n* = 9) and was excellent (ICC = 0.978, *p* < 0.001). In comparing response scores between NDT conditions, performance in the cap condition was significantly lower than the other conditions (open: *t* =  − 4.04, *p* < 0.001, *M* difference =  − 0.93; mesh: *t* =  − 3.65, *p* = 0.002, *M* difference =  − 0.84; extraneous odor: *t* =  − 4.44, *p* < 0.001, *M* difference =  − 1.02). There were no other significant differences between conditions (*p*s ≥ 0.859).

There were no significant effects in the open condition *(p*s ≥ 0.074). However, in the mesh condition*,* there was a significant effect of sex on hit rate, such that males (*M* = 0.779, SEM = 0.074) had greater hit rates than females (*M* = 0.55, SEM = 0.052; *z* = 2.44, *p* = 0.015). There were no other significant effects in the mesh condition (*p*s ≥ 0.173).

**Fig. 3:**
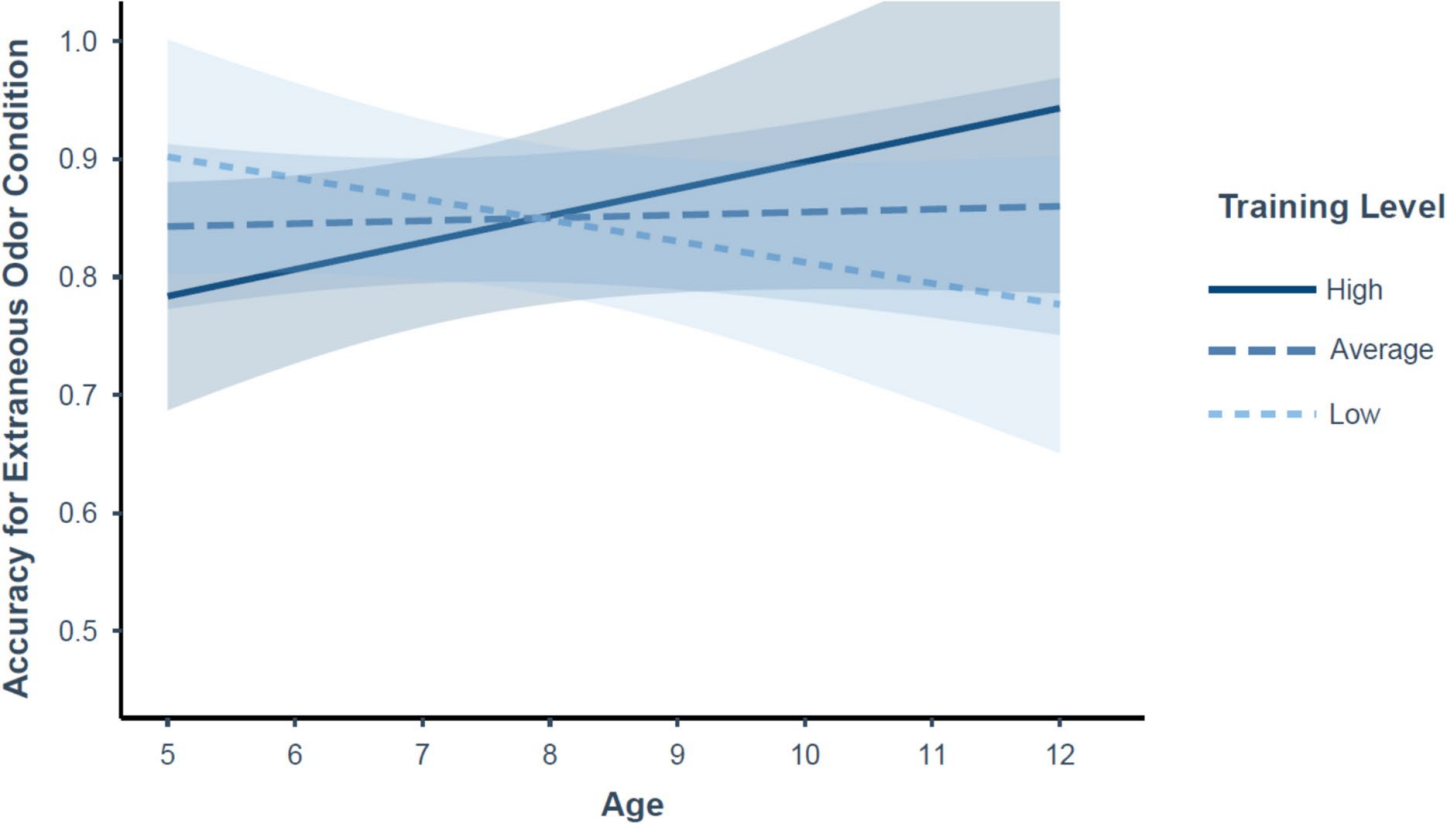
Predicted linear trajectory of accuracy in the extraneous odor condition as a function of age by training. Training was measured as a continuous variable (*M* score = 3.03, SD = 1.07), but for visualization purposes has been plotted relative to the mean value of the sample (dark blue solid line = high training relative to the sample (mean + 1 SD); blue medium dashed line = average training relative to the sample (mean); light blue short dashed line = low training relative to the sample (mean − 1 SD)). Confidence bands represent a 95% confidence interval. Y-axis restricted to observed values

**Fig. 4:**
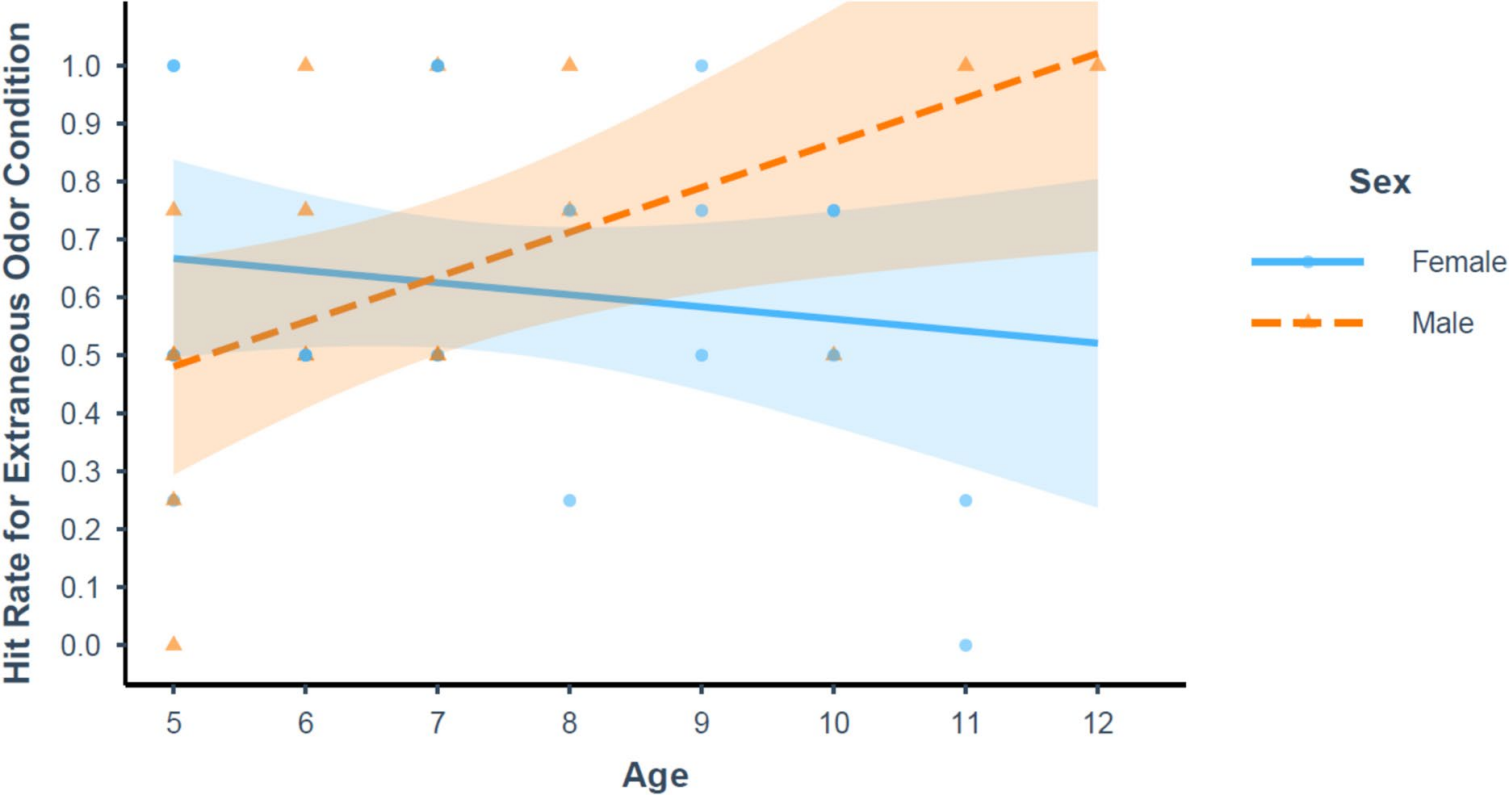
Hit rate in the extraneous odor condition as a function of age for males (orange dashed line) and females (blue solid line). Individual data points are represented by blue circles (females) and orange triangles (males). Confidence bands represent a 95% confidence interval

In the extraneous odor condition, there was a significant age–training interaction on both response scores and accuracy, such that performance increased with age for dogs with greater training levels but decreased with age for dogs with lower training levels (*z* = 2.57, *p* = 0.01; *z* = 2.135, *p* = 0.039, respectively; see Fig. 3). In addition, there was a significant interaction between sex and age on hit rate (*z* = 2.376, *p* = 0.017; see Fig. 4), such that scores increased across age for males (*z* = 2.3106, *p* = 0.021) but were not significantly impacted by age for females (*z* =  − 0.646, *p* = 0.518). There were no other significant effects in the extraneous odor condition (*p*s ≥ 0.257).

In the plastic cap condition, there was a significant effect of training on response scores and hit rate, such that performance increased with greater levels of training (response scores: *z* = 3.354, *p* < 0.001; hit rate: *z* = 2.006, *p* = 0.045). There were no other significant effects in the plastic cap condition (*p*s ≥ 0.109).

Lastly, there was a significant interaction between age and training level on NDT overall response scores, such that performance increased with age for dogs with greater training levels but decreased with age for dogs with lower training levels (*z* = 2.07, *p* = 0.038).

### Cylinder reversal task

Out of the 65 subjects tested, data for 60 subjects was retained for the familiarization phase (*M* score = 1.7, SD = 1.06, range = 1–6), data for 59 subjects was retained for the inhibitory control phase (*M* score = 5.85, SD = 2.33, range = 5–16), and data for 55 subjects was retained for the reversal phase (*M* score = 6.98, SD = 3.21, range = 5–16). For the reversal phase, 49 subjects had complete data concerning the percentage of trials that the dog touched the exterior of the cylinder (per [14]). There was no significant effect of previous cylinder experience on any of the dependent variables nor of first reversal trial scores on reversal performance (*p*s ≥ 0.053), therefore all dogs were combined in the analyses.

There was a significant effect of training level on first reversal trial scores, such that dogs with greater levels of training were more likely to perform the first reversal trial correctly (*z* = 2.23, *p* = 0.026). There was also a significant interaction between sex and training level on reversal phase touches, such that female dogs with greater levels of training exhibited greater percentages of touching (*z* = 3.178, *p* = 0.001), with no effect in males (*z* =  − 0.771, *p* = 441). There were no significant effects of age (*p*s ≥ 0.187).

### Questionnaire

There was complete data for all 65 dogs for PANAS Negative Activation scores (*M* score = 0.468, SD = 0.136, range = 0.218–0.855), Energy and Interest scores (*M* score = 0.797, SD = 0.157, range = 0.35–1), Persistence scores (*M* score = 0.609, SD = 0.174, range = 0.25–1), Excitement scores (*M* score = 0.829, SD = 0.122, range = 0.5–1), and Positive Overall scores (*M* score = 0.729, SD = 0.127, range = 0.42–1). There were significant effects of age, sex, and training level on PANAS Positive Energy and Interest scores, such that scores decreased with age (*z* =  − 2.54, *p* = 0.011), were higher for females (*z* =  − 2.08, *p* = 0.038), and increased with greater levels of training (*z* = 3.10, *p* = 0.002). There were also significant effects of age on the Persistence and Excitement subscales of the PANAS, such that scores decreased with age (*z* =  − 3.12, *p* = 0.002; *z* =  − 3.29, *p* = 0.001, respectively). Age and training level had a significant effect on PANAS Positive Overall scores, such that scores decreased with age and increased with higher levels of training (*z* =  − 3.84, *p* < 0.001; *z* = 2.52, *p* = 0.014, respectively). There were no other significant effects (*p*s > 0.05).

There was complete CCDR data for all 65 dogs (*M* score = 35.2, SD = 3.44, range = 18–47). There were significant effects of age and training level on CCDR scores, such that older dogs displayed higher scores and dogs with higher levels of training displayed lower scores (*z* = 2.02, *p* = 0.048; *z* =  − 2.18, *p* = 0.033, respectively).

### Natural detection task and cylinder reversal task comparison

There were no significant correlations between the NDT and the CRT (*p*s ≥ 0.053).

## Discussion

This study aimed to evaluate the impact of aging on olfactory capabilities in dogs and to determine relationships between declines observed in cognitive and olfactory functions associated with age. The age range in the current study represented mature adult to geriatric age [23] to rule out developmental effects. The capacity of the sample to capture age-related effects was supported by significant relationships observed between age and questionnaire measures previously linked to aging (i.e., CCDR, PANAS). Results of this study demonstrated declines in olfactory performance of dogs with increasing age. However, age effects depended on prior training experience and were only observed under certain conditions, discussed in detail below. Observed age effects support previous findings of age on olfaction reported in dogs [9, 30], but are the first to demonstrate a functional relationship across ages rather than by comparing assigned age groups. Importantly, by measuring age as a continuous factor, the risk of type one error was decreased, and the trajectory of aging was better captured than with categorical evaluations used in previous studies [9]. Additionally, findings by Salamon et al. [9] compared performance of each group to a singular reference group (i.e., young adult dogs). By not comparing all age categories to each other, there was a lack of clarity regarding the aging function, which is important in understanding the trajectory of age-related declines.

Effects of training experience on olfactory detection performance were observed, such that age-related declines in detection accuracy were only observed for dogs with lower levels of training. This result supports previous findings that training experience acts as a neuroprotective buffer against CCD-related cognitive declines [27]. This possible protective effect of training suggests that effects of aging on olfactory performance may not be a concern for working dogs that are engaged in training and work continuously. Similar effects were reported in a recent study, in which age-related declines in Energy and Interest of the PANAS were found for untrained companion dogs, but no age effects were seen for working dogs [31]. However, because training experience was not further examined based on specific type of training, further research is needed to determine whether the nature of the training activity is an important factor in terms of protecting against aging effects. Regardless, the results show that in the absence of any type of training, olfactory detection declines with age.

Interestingly, sex differences were observed for hit rate in the extraneous odor and mesh conditions of the NDT. In the extraneous odor condition, hit rate improved with age for males, but no effect was observed for females. In the mesh condition, there was an overall sex effect where males had greater hit rates than females. These findings were not driven by unbalanced representation of sex in the sample, as the average age between sexes was comparable (see Supplementary Materials). Research on dog sex differences in olfactory capabilities remains ambiguous. Some studies have found greater olfactory activation in females than males [32], some have demonstrated greater detection performance by males compared to females in a working context [33], and others have found no sex effects on olfactory activation [34]. Further, sex differences in CRT motoric self-regulation on the reversal phase (i.e., touch percentages) indicated that females performed worse than males. Thus, NDT sex differences were likely not driven by increasing male inhibitory control levels with age. Although these results align in part with existing literature [33], the cause of these effects is not clear and requires further study.

Current findings also provide context for the conditions under which effects of aging manifest by examining condition-specific NDT performance rather than examination across the entire task as in previous studies [9]. Specifically, age-related reductions in olfactory performance were only revealed in the condition in which dogs were required to detect the target odor among an extraneous odor, a novel condition that was added to the NDT in the current study. This finding indicates that an extraneous odor detection task may be more sensitive to age effects than a detection test with varying levels of odor availability. One possibility is that aging may have greater impacts on olfactory capabilities related to selectivity (i.e., detecting a target odor amidst background “noise”) compared to sensitivity related solely to target odor availability (i.e., odor detection threshold). This observation would have important implications for detection dogs that are required to detect target odors from among a myriad of extraneous odors in the environment, though as noted above this effect was not observed in dogs with more training experience. Alternatively, the condition-dependent findings may be due more broadly to task difficulty not specific to the nature of the olfactory ability, where there may have been ceiling effects for conditions that may not have been challenging enough (e.g., open and mesh conditions) and floor effects for conditions that may have been too difficult even for younger dogs (e.g., cap condition). However, significant differences in performance between conditions were only seen for the cap condition relative to all others, suggesting that the observed effects of aging in the extraneous odor condition were due to the specific capabilities required to solve the task in that condition (i.e., selectivity) rather than the general level of difficulty of the condition. Overall performance also showed an effect of age, but this was likely driven by the extraneous odor condition of the task. This finding is the first to demonstrate an effect of age on selectivity, revealing insights into the effects of aging under varying task demands.

NDT completion scores (i.e., completion of all stages of the task, regardless of detection accuracy) were significantly impacted by age and training level, such that dogs with greater training levels were more likely to complete the NDT and older dogs were less likely to complete the NDT. The completion measure was likely measuring physical and motivational variables such as fatigue, task comprehension, and engagement rather than detection ability performance, and was assessed to ensure that such factors did not confound olfactory effects of interest in the NDT. As a result, 21 dogs either did not advance to the NDT or did not complete the entirety of the NDT. This both maintained the integrity of the NDT (i.e., it helped to isolate olfactory capabilities) and revealed potential impacts of other physical (e.g., decreased motor function) and cognitive (e.g., motivation) factors on performance. The impact of aging on NDT completion suggests that older dogs experience age-related declines in areas such as physical capabilities and processes related to motivation, in line with prior findings [27]. Observations emphasize the need to include a warmup phase and termination criteria when evaluating olfactory capabilities using a task that is influenced by physical and motivational factors such as the NDT. Without their inclusion, performance on the NDT could be partially shaped by dogs’ cognitive processes, their physical condition, and learning of the task setup. The impact of training level suggests that, while the task was intended to not require prior training, motivation for and learning of the task can still be influenced by past experiences. This training effect is similar to results of Salamon et al. [10], in which training resulted in a greater likelihood of reaching higher levels of the task. However, it is unclear whether advanced training leads to task completion benefits, or if dogs exhibiting certain traits (e.g., motivation, trainability) are more likely to excel in training, with those same characteristics being beneficial to task completion.

Beyond the NDT, training level significantly impacted CRT performance, and both age and training level impacted many of the measures assessed in the cognitive and behavioral questionnaire scales. Although no dogs demonstrated diagnosable levels of CCD, the CCDR showed a significant effect of age and training level, such that older dogs demonstrated greater symptomology of CCD, and a greater training level was associated with decreased CCDR scores. Training level also resulted in better initial performance on the CRT reversal phase. As discussed above, these findings support recent studies that found that training plays a protective role against age-related declines [27], encouraging further exploration of this topic. Age was also significantly associated with cognitive and behavioral decline as assessed by multiple subscale measures of the PANAS, indicating decreases in measures of energy due to age and training level. These effects provide further evidence that aging and experience affect cognitive and behavioral function and validate the observation that the age range of the sampled population in the study was adequate to capture effects of interest. The effects of aging on the PANAS directly replicate previous findings of decreased positive activation in dogs with age [28].

Contrary to Hargrave et al. [14], there were no significant age effects on CRT performance. These differing results could be attributed to multiple factors. The findings of Hargrave et al. [14] were the result of a principal component analysis, while our findings analyzed multiple task performance measures. As a result, the findings of Hargrave et al. [14] reflect an overall combined measure of performance, while current results reflect individual effects, making direct comparisons difficult and perhaps leading to variability in results. In addition, there were slight differences in methodologies of the CRT between the two studies. Most notably, Hargrave et al. [14] conducted a fixed number of reversal trials (8) whereas this study used a performance criterion (i.e., 4/5 correct reversal trials) to determine the total number of reversal trials. Thus, it is possible that this performance criterion was not stringent enough, and additional trials may have revealed effects. However, some of the current CRT findings are supportive of prior studies, such as the lack of a training effect in the inhibitory control portion of the task [35].

Contrary to the hypotheses, overall NDT accuracy and overall CRT performance were not correlated. However, inhibitory control only represents one cognitive domain. Furthermore, because age effects were not found for the CRT, whether age effects on these two tasks are correlated could not be properly examined. Due to time limitations, a single, rapid task was selected to measure aspects of cognition. The CRT was selected due to previous findings that the CRT is associated with decreases in cognition as measured via survey, indicating that performance on this task can be linked to overall cognitive declines [14]. In light of this and the lack of similar effects in our sample, further examinations of the relationship between declines in cognition and olfaction are warranted.

Although this study reveals important information regarding dog aging processes, there are limitations. While the sample size was large for a dog experimental study [36], owners that participate in training activities with their dogs are likely more willing to participate in research, and thus there was a large number of dogs with greater training. In the future, a more even distribution of training experiences would be preferred. Additionally, we did not examine specific types of training experience due to uneven distribution of dogs with certain types of training, which will be important in future studies to elucidate the training effects. Furthermore, older dogs were more likely to fail to complete all phases of testing, biasing the sample toward the younger end of the target range. While recruitment was targeted toward older dogs, factors such as decreased physical capabilities and lifespan limitations impacted collection of data on dogs near the end of their lifespan. Thus, it is likely that additional age effects would have been observed had a greater number of senior and geriatric dogs been included, highlighting an important consideration in research focused on canine aging. Additionally, complete data were not collected on all subjects. While rigorous termination criteria prevented the NDT from being impacted by factors such as motivation, it resulted in a substantial portion of the sample failing to complete the task. As a result, comparisons between NDT performance and CRT performance could only be made with a partial, and potentially skewed, subset of the data. It should also be noted that while the open condition of the NDT was always conducted first as a baseline condition, whether lack of effects on this level was due to its primacy or its ease cannot be untangled. Despite these limitations, this study provides novel information concerning age-related declines in dog olfaction and, to our knowledge, is the first study to examine relationships between cognitive and olfactory declines in dogs.

## Conclusion

The current study found that aging impacted olfactory capabilities, but these effects were condition-dependent (e.g., aging effects were only observed in the extraneous odor condition). Training level further modulated aging effects, supporting previous findings of training serving as a neuroprotective factor. These findings suggest that aging can influence dog olfactory performance, but that the effect of aging may be dependent upon task parameters and shaped by interactions with other variables (e.g., sex, training). Future studies should aim to continue to examine age functions for different processes in the dog, as well as consider the effects that motivation and task parameters can have upon dogs’ performance of the NDT.

## Supplementary information

Below is the link to the electronic supplementary material.

Supplementary file (PDF 157 KB)

## Data Availability

Access to data files is available upon request from the corresponding author via email (lim0004@auburn.edu).
