# Supplementary material for: Evaluating effects of aging on dog olfactory performance
Source: GeroScience. 2025 Nov 3;48(1):17–30. doi: 10.1007/s11357-025-01905-1 (PMC12972148; doi:10.1007/s11357-025-01905-1)
Supplement: Supplementary file 1 — Supplementary file (PDF 157 KB) [file 11357_2025_1905_MOESM1_ESM.pdf]

**Supplementary Information**
**Title:** Evaluating Effects of Aging on Dog Olfactory Performance
**Journal:** GeroScience
**Authors:** Lane I. Montgomery*, Sarah Krichbaum, Jeffrey S. Katz, and Lucia Lazarowski
*Department of Psychological Sciences, Auburn University, lim0004@auburn.edu

**Table S1. Breed information for all dogs included in the study**

| Breed | Number | Males | Females |
|---|---|---|---|
| Australian Shepherd | 1 | 0 | 1 |
| Boykin Spaniel | 1 | 1 | 0 |
| Catahoula Leopard Dog | 2 | 0 | 2 |
| Chihuahua | 1 | 0 | 1 |
| Dutch Shepherd | 1 | 1 | 0 |
| English Shepherd | 1 | 1 | 0 |
| Flat-Coated Retriever | 1 | 1 | 0 |
| Foxhound | 1 | 1 | 0 |
| French Bulldog | 1 | 0 | 1 |
| German Shepherd | 9 | 2 | 7 |
| German Shorthaired Pointer | 1 | 0 | 1 |
| Golden Retriever | 1 | 1 | 0 |
| Jack Russell Terrier | 1 | 1 | 0 |
| Labrador Retriever | 16 | 9 | 7 |
| Shiba Inu | 1 | 1 | 0 |
| Siberian Husky | 1 | 1 | 0 |
| Standard Poodle | 3 | 1 | 2 |
| Weimaraner | 1 | 0 | 1 |
| Mixed Breed | 21 | 3 | 18 |

**Table S2. Age Distribution by Sex for the NDT**

| Sex | Number of Dogs | Mean Age | SD |
|---|---|---|---|
| M | 17 | 7 | 2.18 |
| F | 25 | 7.36 | 2.02 |
